# Factors Influencing Admission Decisions in Skilled Nursing Facilities: Retrospective Quantitative Study

**DOI:** 10.2196/43518

**Published:** 2023-05-17

**Authors:** Caroline Strickland, Nancy Chi, Laura Ditz, Luisa Gomez, Brittin Wagner, Stanley Wang, Daniel J Lizotte

**Affiliations:** 1 Department of Computer Science University of Western Ontario London, ON Canada; 2 PointClickCare Mississauga, ON Canada; 3 Department of Epidemiology and Biostatistics University of Western Ontario London, ON Canada

**Keywords:** decision-making, skilled nursing facility, patient admission, decision, nursing, clinical, database, health informatics, diagnosis, modeling, connection, patient

## Abstract

**Background:**

Occupancy rates within skilled nursing facilities (SNFs) in the United States have reached a record low. Understanding drivers of occupancy, including admission decisions, is critical for assessing the recovery of the long-term care sector as a whole. We provide the first comprehensive analysis of financial, clinical, and operational factors that impact whether a patient referral to an SNF is accepted or denied, using a large health informatics database.

**Objective:**

Our key objectives were to describe the distribution of referrals sent to SNFs in terms of key referral- and facility-level features; analyze key financial, clinical, and operational variables and their relationship to admission decisions; and identify the key potential reasons behind referral decisions in the context of learning health systems.

**Methods:**

We extracted and cleaned referral data from 627 SNFs from January 2020 to March 2022, including information on SNF daily operations (occupancy and nursing hours), referral-level factors (insurance type and primary diagnosis), and facility-level factors (overall 5-star rating and urban versus rural status). We computed descriptive statistics and applied regression modeling to identify and describe the relationships between these factors and referral decisions, considering them individually and controlling for other factors to understand their impact on the decision-making process.

**Results:**

When analyzing daily operation values, no significant relationship between SNF occupancy or nursing hours and referral acceptance was observed (*P*>.05). By analyzing referral-level factors, we found that the primary diagnosis category and insurance type of the patient were significantly related to referral acceptance (*P*<.05). Referrals with primary diagnoses within the category “*Diseases of the Musculoskeletal System*” are least often denied whereas those with diagnoses within the “*Mental Illness*” category are most often denied (compared with other diagnosis categories). Furthermore, private insurance holders are least often denied whereas “*medicaid*” holders are most often denied (compared with other insurance types). When analyzing facility-level factors, we found that the overall 5-star rating and urban versus rural status of an SNF are significantly related to referral acceptance (*P*<.05). We found a positive but nonmonotonic relationship between the 5-star rating and referral acceptance rates, with the highest acceptance rates found among 5-star facilities. In addition, we found that SNFs in urban areas have lower acceptance rates than their rural counterparts.

**Conclusions:**

While many factors may influence a referral acceptance, care challenges associated with individual diagnoses and financial challenges associated with different remuneration types were found to be the strongest drivers. Understanding these drivers is essential in being more intentional in the process of accepting or denying referrals. We have interpreted our results using an adaptive leadership framework and suggested how SNFs can be more purposeful with their decisions while striving to achieve appropriate occupancy levels in ways that meet their goals and patients’ needs.

## Introduction

### Background

More than 15,100 skilled nursing facilities (SNFs) in the United States provide care for over 1.4 million patients [1]. These SNFs are multidisciplinary health care settings where patients receive short- and long-term skilled services from rehabilitation, nursing, and other care disciplines. The patients within these facilities may have a variety of conditions, comorbidities, and disabilities, but they are stable to be discharged from the hospital.

Occupancy rates within these SNFs have been decreasing since the early 1980s owing to substantial growth in the assisted living and residential care field and have been declining at an even higher rate since the beginning of the COVID-19 pandemic [2]. With decreasing occupancy rates comes an increase in the number of beds within these SNFs that are left unoccupied.

Low occupancy is a financial challenge for facilities that bear fixed costs for unoccupied beds; hence, there is an incentive to maintain or increase occupancy levels, subject to operational constraints such as staffing and the ability to provide the required specialized care.

Occupancy in SNFs is determined by patient outflow through discharge and inflow through accepted referrals. An SNF admissions coordinator may decide to offer a bed to a referred patient if they are interested in adding the patient to their roster and case mix, or they may decide to deny the referral if they feel the patient is not a good match for the facility at that time because of financial, clinical, or operational considerations. Balancing facility resources and capacities to meet patient needs may traditionally be viewed as a straightforward process [3]. However, dramatic policy changes impacting where hospitals discharge medically complex patients together with the range of services that patients may receive in post–acute care settings [4] present a complicated, dynamic context calling for decision makers at SNFs to address blind spots and work together to address the dynamic challenges that these organizations face.

**Figure 1:**
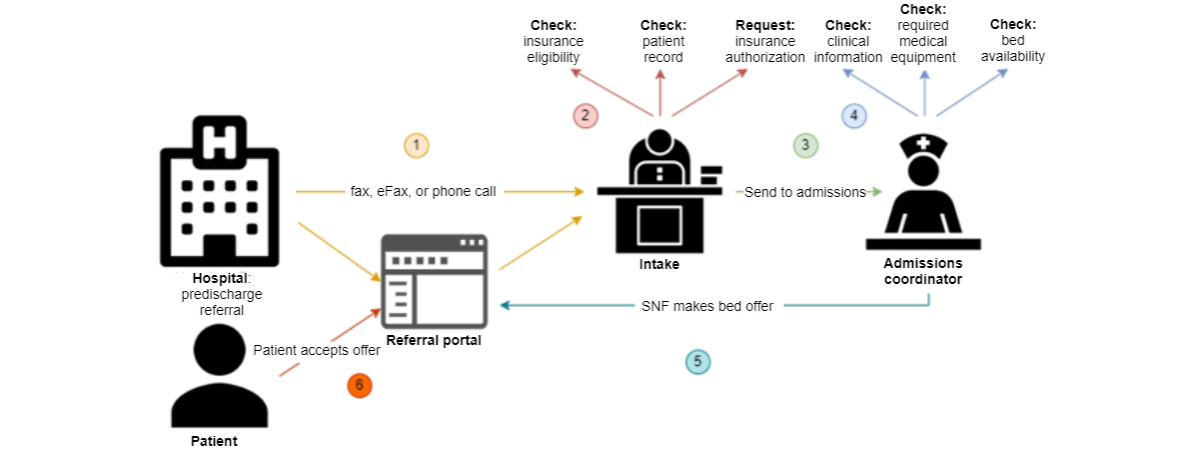
Skilled nursing intake flow. The circled numbers denote the temporal ordering of a referral, starting at the hospital and ending with a patient accepting an offer from a nursing facility. SNF: skilled nursing facility.

If an offer is made, the patient and their carers may decide to accept it and go to that SNF for care. The steps and associated information flow involved in the referral process are illustrated in Figure 1. This process begins at the hospital via a predischarge referral, where information about the patient, including health information and financial arrangements, is either shared electronically or manually sent to a discharge planner via phone or fax. The discharge planner then sends the referral to a set of potential facility admissions coordinators who check if their facility can meet the needs of the patient. If, after analyzing all available information, the admissions coordinator decides not to offer the patient a bed, the referral is denied and no offer is sent back. Otherwise, the admissions coordinator sends an offer either electronically or manually, at which point the patient can accept or refuse the offer. It is common for a hospital to send several referrals to different SNFs for the same patient, simultaneously or in quick succession; hence, it is of utmost importance for admissions coordinators to provide a timely response with an offer if their goal is to increase occupancy. Not only do they stand to lose the referral to competing SNFs if they are slow to respond, but also hospitals measure referral response times; if an SNF has consistently late responses, it can impact whether future referrals are sent to that facility.

In this study, we provide the first comprehensive analysis of financial, clinical, and operational reasons that differ between referrals that impact whether a referral is accepted or denied by an SNF. To do this, we summarized and analyzed records from a large database of referrals extracted from an integrated health informatics system, including both referral-level information and facility-level information.

Our key contributions were as follows:

1. A description of the distribution of referrals in terms of key referral- and facility-level features.
2. An analysis of the relationship among key financial, clinical, and operational variables and rates of denial.
3. A discussion identifying the key potential reasons behind referral decisions in the context of learning health systems.

Our work is the first large-scale quantitative analysis of referrals to SNFs and associated offer or denial decisions and represents a first step toward providing sequential decision support to personnel managing SNF admissions.

### Prior Work

Previous studies focused on clinical variables alone have found that patients with characteristics associated with faster recovery and discharge to the community are more likely to be referred to and accepted into hospital-based post–acute care units or hospital-based SNFs, and these studies also highlight implications for patient and facility outcomes [5-7].

Zimmer et al [8] stated that “while we may expect the most disabled, sick, and elderly patients to be the primary occupiers of beds within our nursing homes, this is not the case. As death often cuts their stays short, we end up seeing a higher composition of patients with complex conditions and a greater life expectancy” [9]. This “typical” resident is described as older adult, female, and having multiple impairments in their activities of daily living [10]. Other studies have delved deeper into describing commonly appearing traits of this population. Caffrey et al [11] revealed that in 2010, nearly 2 in 10 residents were medicaid beneficiaries, and almost 6 in 10 residents aged ≤65 years had medicaid. Furthermore, almost 4 in 10 residents received assistance with ≥3 activities of daily living (of which bathing and dressing were the most common). Finally, more than three-fourths of residents have had at least 2 of the 10 most common chronic conditions (high blood pressure, Alzheimer disease, and other dementias) [11].

These and other works [8,12] have enhanced our understanding of the composition of our population residing in SNFs as well as specific characteristics associated with referral acceptance. The gap that currently exists in previous research is a holistic analysis of the patterns of decision-making when it comes to referral decisions being made by SNFs. We wanted to understand, on a macro level, what types of features play into whether an SNF ultimately accepts or denies a referral. This includes looking at not only one type of diagnosis or insurance but many. It also involves considering daily operational information such as occupancy and nursing hours as well as facility characteristics such as geographic location and overall 5-star rating. The goal of our work was to fill this gap and offer answers to the high-level question, “What are the factors that play a significant role in an SNF deciding to accept or deny a referral from the hospital?”

## Methods

In this section, we describe the data sources, preprocessing steps, and descriptive and analytical methods used in this study.

### Data Sources and Preprocessing

Our primary data source contains over 1.5 million referral records from 5218 SNFs, from which we have collected data within the United States. This represents a little over a third of all SNFs operating in the United States between January 1, 2020, and March 31, 2022 (inclusive), the range of dates we considered in our analyses.

A subset of the facilities that generated these data used a software module to simplify the referral process by automating communications with referrers—referrals from hospitals are automatically inserted into the SNF’s database, saving time that would otherwise be used for data entry. Thus, this software ensures that every referral to the SNF is recorded in the database, regardless of the offer or denial decision and regardless of whether the patient is ultimately admitted to the SNF. We termed a referral that is admitted to an SNF as a “win,” a referral on which an offer is made that does not lead to admission as a “loss,” and a referral that the SNF rejects as a “denial.” In Multimedia Appendix 1 we can see that facilities not using electronic referral software are logging fewer losses and denials than those that are. On the basis of the consultation with subject-area experts, we believe that this is because a major portion of losses and denials are not being recorded manually owing to the additional effort required to enter that information manually, as loss and denial information is less critical for day-to-day facility operations. Thus, we have restricted our analysis to referrals where referral automation was used, as these should provide a view of referral decision-making that is not biased against losses and denials and reduces variation in data collection methods at the facility level.

Filtering based on automated referral software and insurance type resulted in *179,150* referral records from *627* facilities across *41* states in the United States.

These records can be used to extract 3 types of information associated with each referral; for denied referrals only, there is additional information from a field denoting the *reason* for the denial, set by the admissions coordinator. the top 3 recorded reasons are “issues with patient (high risk or behavioral issues)” (2149/6802, 31.59%), “lack of clinical resources” (1952/6802, 28.7%), and “financial issues” (1822/6802, 26.79%); while this provides some insights into denials, it has very high missingness (4906/11,708, 41.9% of all referrals) or is set to “other,” and it cannot be used to compare denied referrals with accepted referrals. Hence, for our analysis, we will instead use more complete information about patient finances, primary diagnosis, and facility operations information):

1. Daily operational information about the receiving SNF that changes daily and is linked to referrals temporally.
2. Clinical and financial properties intrinsic to the referral that do not change, regardless of what SNF the referral was sent to.
3. Characteristics of the receiving SNF that do not change or that change on much longer timescales than daily operational data. These data are collected at the time of referral.

### Ethics Approval

The study, along with a waiver of consent for secondary analysis of deidentified data, was approved by the Western University Health Sciences Research Ethics Board under project ID 114989. The Western University Health Sciences Research Ethics Board operates within the provisions of the Ontario Personal Health Information Protection Act (2004) and its applicable regulations and is registered with the US Department of Health & Human Services under institutional review board registration number institutional review board 00000940.

### Daily Operations Data

Each referral record is tagged with a recipient Centers for Medicare and Medicaid Services Medicare number, which allows us to identify which referrals flow to each facility and to associate facility-level information with referrals. For each referral, we calculated both an occupancy level of the SNF that received the referral (calculated at the time the referral was sent) and a facility self-reported collection of patient hours for unique types of nurses. Occupancy is computed daily using facility data representing both the current number of beds filled on a given day and the total number of beds in each facility; we divided the latter by the former and multiplied by 100 to obtain the percentage occupancy for that day.

We note that this measure of occupancy is approximate and subject to noise; this is explained more fully in the Limitations section.

Next, to acquire nursing hour information, we gathered all documented schedules of the facility in question and paired them with the referral data based on the day the referral was sent out. These scheduling data are retrieved from the payroll-based journal data set [13] and include all hours worked for registered nurses (RNs), licensed practical nurses (LPNs), and certified nursing assistants (CNAs). We then divided these hours by the number of occupied beds in the facility at the specified date to retrieve the nursing hours per patient. We noted that this measure of nursing hours per patient is also approximate and subject to noise, as the divisor (occupied beds) may vary slightly depending on the time of day in which the value was recorded.

### Patient Characteristic Data

We extracted the insurance type associated with each referral as categorical variables, including Medicare Advantage (we will be referring to this as “medicare A” throughout our paper), Medicare B, managed care, medicaid, outpatient, private, and other. Percentage distributions of these insurance types are shown in Multimedia Appendix 2. We omitted Medicare B and Outpatient referrals from our analysis because these records were rare (261/179,150, 0.14%).

**Figure 2:**
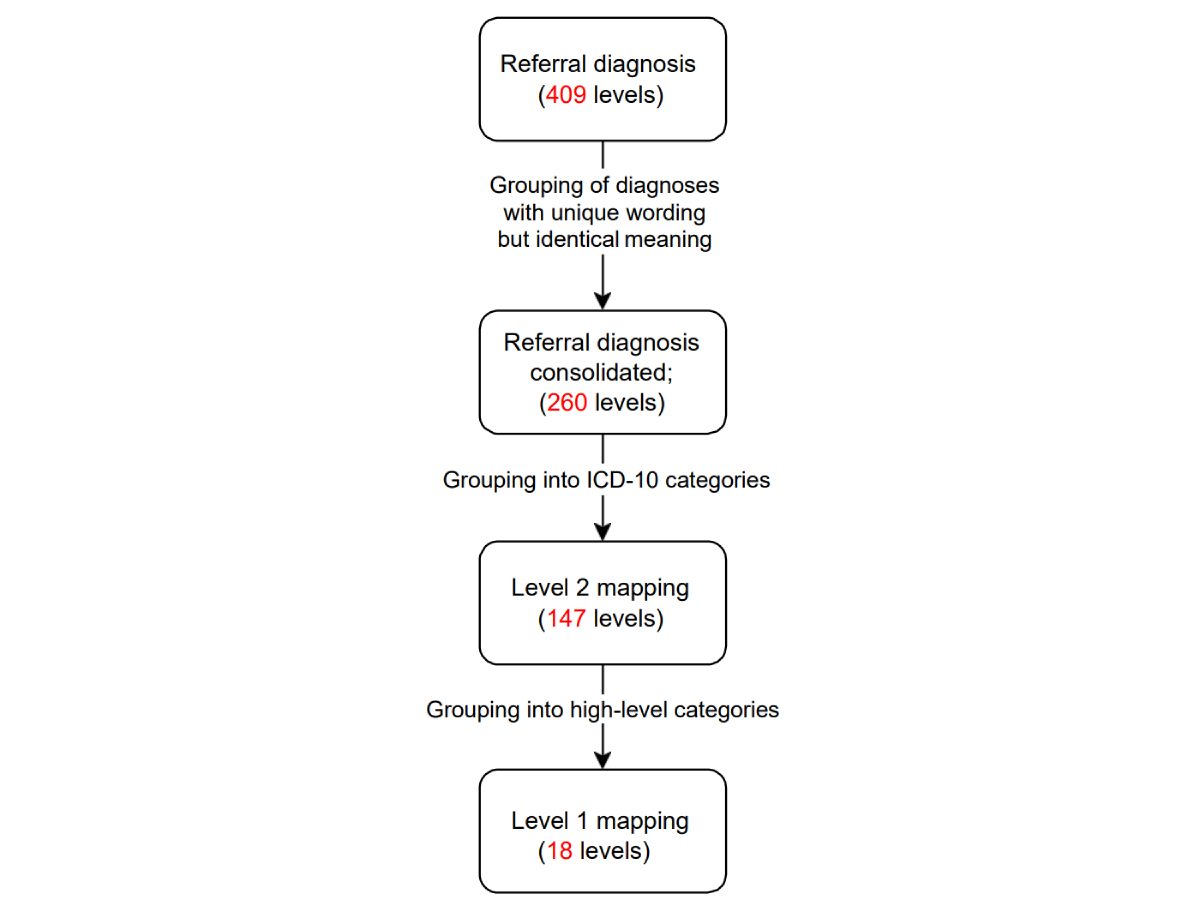
Path of grouping primary diagnosis levels within referral data. ICD-10: International Classification of Diseases 10th Revision.

More than 400 unique “primary diagnosis” types, entered by the hospital, are represented in referral data as patient characteristics. The primary diagnosis represents the most pressing medical issue for which the patient is referred. The primary diagnosis can be entered manually, and because of this, there are numerous diagnoses that are unique in spelling but identical in denotation; for example “hip fx” and “hip fracture.” To manage this issue, we first combined all diagnoses that have unique wording or spelling but are identical in meaning. This reduced the number of unique categories from 409 to 260. We then manually created a mapping that combined the remaining 260 diagnosis types into broader “buckets” or categories. This mapping is analogous to that used by the Clinical Classification Software for the International Classification of Diseases, 10th Revision (ICD-10) [14], which is used for classifying diagnoses and reasons for visits in health care settings. After consolidating the referral primary diagnosis levels in a many-to-one fashion, we manually aggregated each of the 260 unique diagnosis types into 1 of 147 different ICD-10 codes. It is important to note here that this is a subset of all ICD-10 codes, as we only included those populated by at least 1 of our referral diagnoses. From there, each of the sorted codes is rolled up into 18 more abstract diagnosis categories based on the Clinical Classification Software Level 2 labels [14]. These categories are listed in Table 1, and a figure visualizing the aforementioned process is shown in Figure 2.

**Table 1:** Mapping of category value to diagnosis definition.

Category	Definition
1	Infectious and Parasitic Diseases
2	Neoplasms
3	Endocrine, Nutritional, and Metabolic Diseases and Immunity Disorders
4	Diseases of the Blood and Blood-Forming Organs
5	Mental Illness
6	Diseases of the Nervous System and Sense Organs
7	Diseases of the Circulatory System
8	Diseases of the Respiratory System
9	Diseases of the Digestive System
10	Diseases of the Genitourinary System
11^a^	Complications of Pregnancy; Childbirth; and the Puerperium
12	Diseases of the Skin and Subcutaneous Tissue
13	Diseases of the Musculoskeletal System and Connective Tissue 14
14^a^	Congenital Anomalies
15^a^	Certain Conditions Originating in the Perinatal Period
16	Injury and Poisoning
17	Symptoms and Ill-Defined Conditions Influencing Health Status
18	Residual Codes (Unclassified)

^a^Does not appear within the filtered data sample.

### Facility Characteristic Data

For each referral, we extracted the overall 5-star rating associated with receiving SNF as categorical variables ranging from 1 to 5 stars. In general, a 1-star rating corresponds to facilities that have a much below–average quality, whereas a 5-star rating corresponds to facilities that have a much above–average quality. Multimedia Appendix 3 provides a detailed distribution of the overall 5-star ratings.

In addition, for each referral, we extracted the geographic area (whether the facility resides in an urban or rural area) of the receiving SNF as categorical variables. A detailed distribution of geographic areas can be found in Multimedia Appendix 3.

### Description and Analysis Methods

To understand the referral- and facility-level factors that contribute to whether an offer is made on a referral, we first collected and presented summary statistics of our data set. We then constructed a series of logistic regression models relating these factors to offer decisions. Our outcome of interest was “acceptance of referral” (AOR), which is 0 for a referral if a patient is not offered a bed (denied) and 1 if the patient is offered a bed (won or lost). All analyses were performed using a combination of the GLM package in R (version 4.0.5; R Foundation for Statistical Computing) and the Statsmodels library for Python (version 3.10.5).

### Daily Operations

We first examined the relationship between occupancy and referral denials by running a univariate logistic regression of AOR on “occupancy tertile” (low, medium, or high, relative to the occupancies of all referrals in the data set). We then regressed AOR on the 3 quantitative nursing hours per patient variables (CNA, LPN, and RN) as predictors in a multivariate model.

### Patient Characteristics

Next, we examined how the characteristics of the patient being referred relate to AOR, focusing on primary diagnosis and insurance type. We constructed three logistic regression models: (1) regressing AOR on the primary diagnosis mapping of the patient being referred, (2) regressing AOR on the insurance type of the patient, and (3) regressing AOR on both variables together to better understand their relative contributions when each is adjusted for the other. We elected not to adjust for daily operations features in our analyses because of data quality concerns, which is discussed further in the Limitations section of this paper.

### Facility Characteristics

In our final analyses, we examined the relationships between 2 facility characteristics: overall 5-star rating and rural versus urban status and AOR. First, we regressed AOR on the overall 5-star rating of the facility receiving the referral. The overall 5-star rating is composed of quality measure scores, staffing, and survey results and is recalculated multiple times per year. For our study, we used the calculated overall 5-star rating at the time of referral. We then regressed AOR on the geographic area to investigate any differences. We then fit a multivariate model by regressing AOR on both simultaneously. We did not adjust for daily operations features in our analyses because of nonsignificance as well as data quality concerns, which is discussed further in the Limitations section of our work.

## Results

In the results, we identified several significant differences between AORs for different groups of patients and SNFs. Although the evidence for differences in AORs is strong, as expected, none of the models are highly predictive at the level of individual referrals, as they were not intended to capture all of the many factors that may influence an individual referral.

### Data Summaries

**Figure 3:**
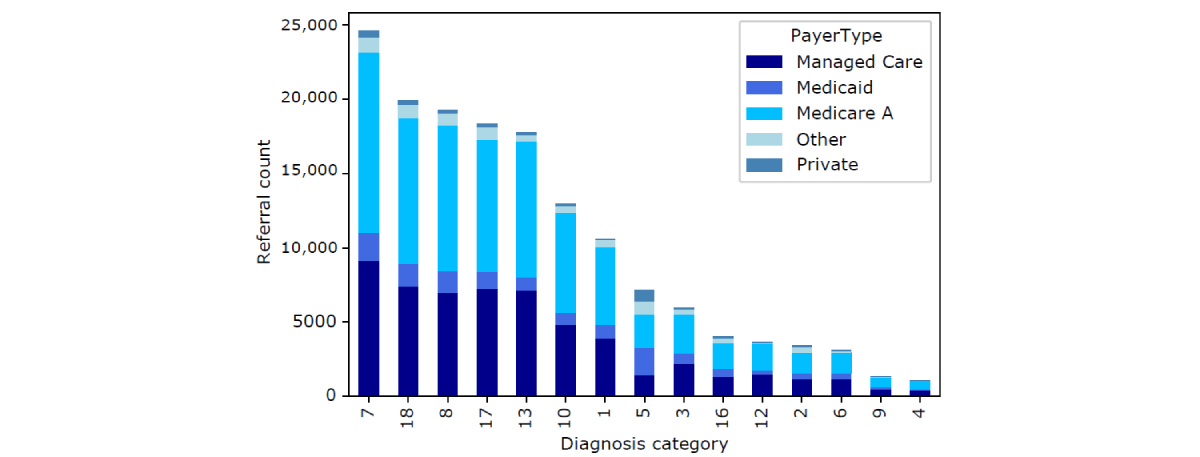
Counts of referral diagnosis types and their associated insurance composition.

**Figure 4:**
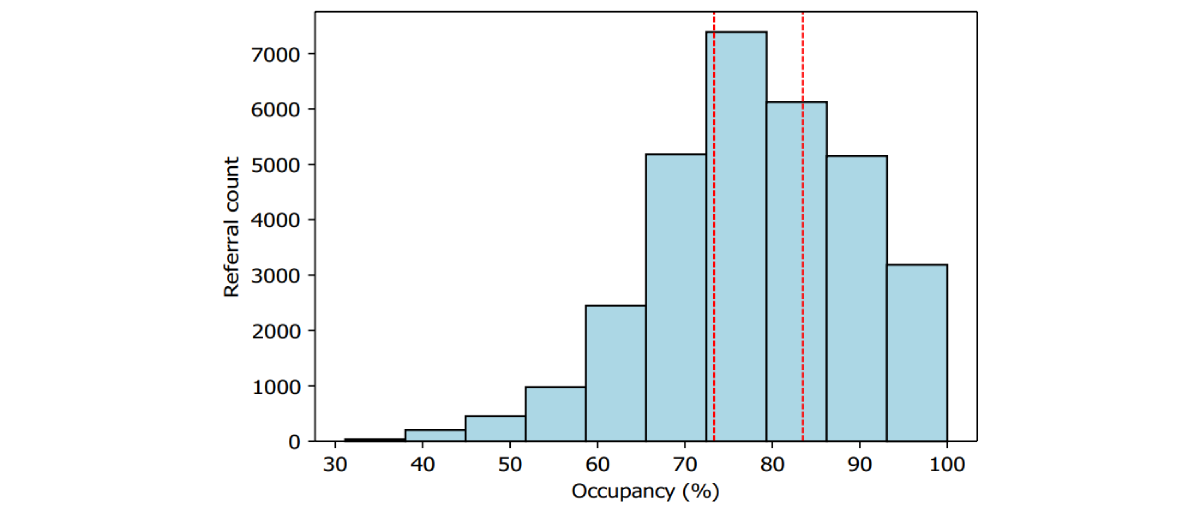
Occupancy of facilities upon receiving a referral. Each portion (separated by the vertical red dotted lines) signifies which quartile the corresponding occupancies belong to. The leftmost portion is "low" (0%-73.3%), the middle portion is "mid" (73.4%-83.5%), and the rightmost portion is "high" (83.6%-100%).

**Figure 5:**
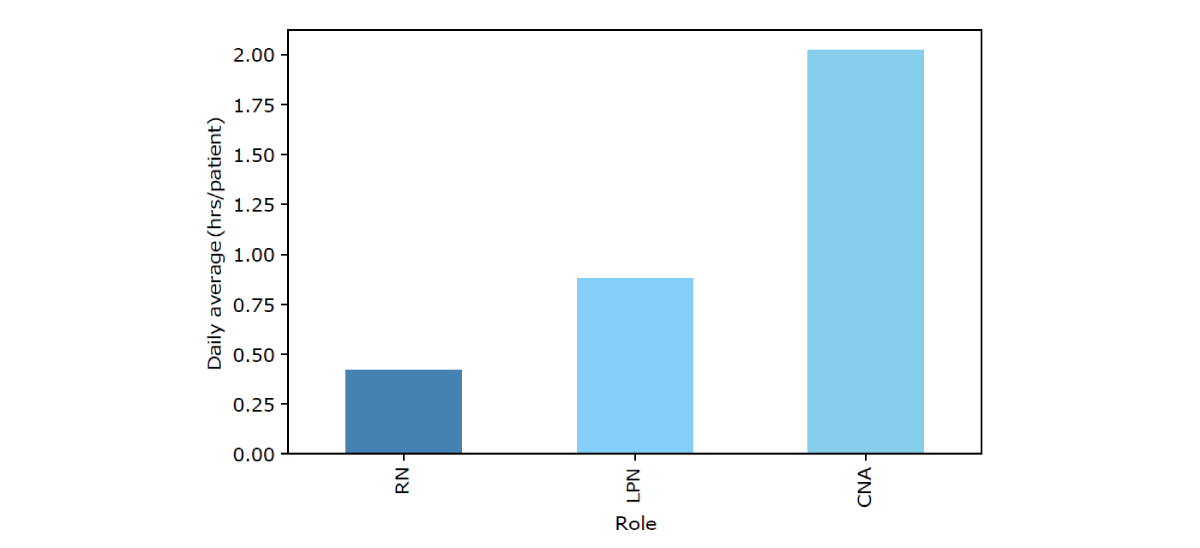
Overall averages for nursing hours per day in a facility. The x-axis comprises registered nurses (RNs), licensed practical nurses (LPNs), and certified nursing assistants (CNAs).

**Figure 6:**
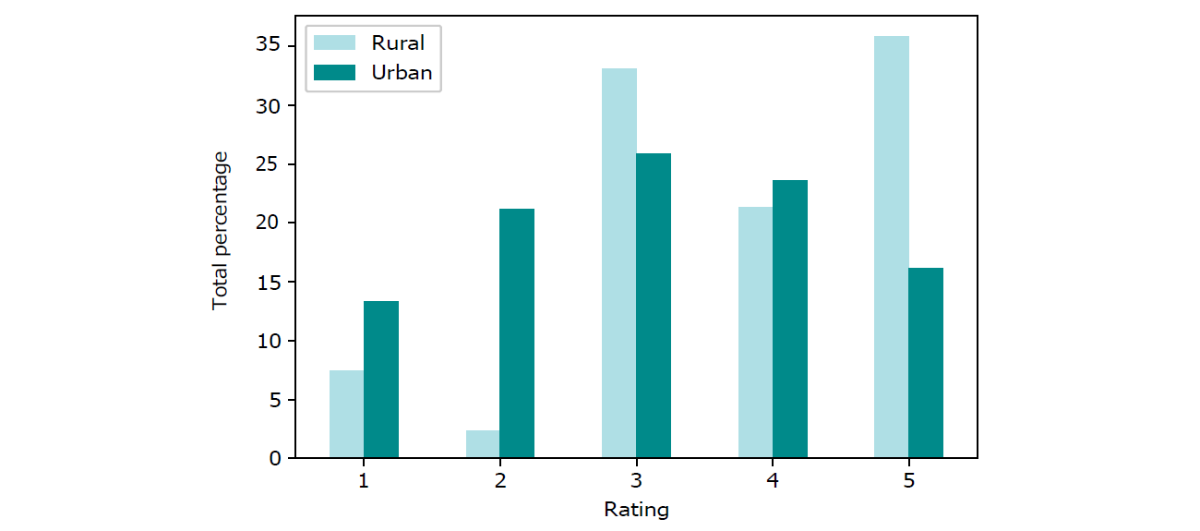
Breakdown of overall ratings within rural (54 unique facilities and 5572 referrals) versus urban (570 unique facilities and 173,578 referrals) facilities.

As discussed previously, our data consist of 179,150 referrals in which there exist 5 primary types of insurance, 15 distinct diagnosis categories (as 3 of 18 diagnosis categories did not appear as data points within our data), and occupancy and nursing hours at the time of referral. In total (167,442/179,150, 93.46%) of referrals were met with SNF offers. The composition of both insurance type and diagnosis categories within this data is shown in Figure 3, with a more detailed summary provided in Multimedia Appendix 2. The composition of occupancy percentages is shown in Figure 4, where each of the 3 vertical perimeters represent tertiles ranging from low, medium, to high occupancy. A breakdown of total nursing hour averages per day are shown in Figure 5. Finally, the composition of facility-level features is shown in Figure 6 with a more detailed summary provided in Multimedia Appendix 3.

### Regression Analyses—Daily Operations Occupancy and Nursing Hours

We first analyzed the impact of occupancy on AOR. On the basis of the results of the model previously described, no statistically significant difference in AOR was found between facilities with a “high” occupancy compared with our reference level of “mid” occupancy (95% CI for log odds ratio −0.162 to 0.018), and no statistically significant difference was found for AOR comparing facilities with a “low” occupancy compared with our reference level of “mid” occupancy (95% CI for log odds ratio −0.028 to 0.158).

Next, we analyzed the impact of nursing hours on AOR. To account for the increased variability from facility to facility, we implemented a mixed effects model and introduced random effects (intercept and slope) to a multivariate model. It is important to note that the highest correlation coefficient is 0.349 (between CNA hours per patient and RN hours per patient), so we can rule out suspicions of colinearity in our model. The results of this model can be seen in Table 2. There was a small increase in AOR, given a 1-hour increase in CNA and LPN hours per patient (0.3% and 1.2%, respectively). Conversely, there was a small decrease in AOR, given a 1-hour increase in RN hours per patient (1.1%). Thus, there appeared to be no statistical significance in the relationship between nursing hours and AOR within our data, and so no conclusive statements can be made.

As both occupancy and nursing hours appear to be nonsignificant and, as discussed in the Methods section, may not be accurate at the time of referral, we left these features out of our referral- and facility-level analyses as possible confounding variables.

**Table 2:** Logistic regression results for nursing hours.

	Coefficient	SE	*z* value	*P* value	95% CI
Intercept	0.89	0.020	44.4	<.005	0.85 to 0.93
CNA^a^	0.003	0.011	0.241	.81	−0.019 to 0.024
RN^b^	−0.011	0.021	−0.517	.61	−0.053 to 0.031
LPN^c^	0.012	0.015	0.813	.42	−0.018 to 0.0

^a^CNA: certified nursing assistant.

^b^RN: registered nurse.

^c^LPN: licensed practical nurse.

### Regression Analyses—Patient Characteristics Diagnosis

A significant association was observed between diagnosis and AOR. The detailed results of this model are listed in Table 3. It is important to note that this model uses category 7 (Diseases of the Circulatory System) as the reference level. In cases similar to this study, where there is no normative group based on theory, it is customary to choose the largest level as the reference, which in this case is diseases of the circulatory system, as shown in Multimedia Appendix 2. This means that the results of all other primary diagnosis types are computed relative to category 7, whose AOR-predicted probability was 94.7%.

**Table 3:** Logistic regression results for primary diagnosis type (reference level—Diseases of the Circulatory System).

Diagnosis	Coefficient	SE	*z* value	*P* value	95% CI
Intercept	2.88	0.03	101	<.005	2.82 to 2.93
1	−0.199	0.05	−4.07	<.01	−0.294 to −0.1
2	−0.008	0.081	−0.1	.92	−0.17 to 0.15
3	−0.32	0.06	−5.64	<.005	−0.44 to −0.21
4	0.13	0.15	0.89	.38	−0.156 to 0.416
5	−0.88	0.046	−19.0	<.005	−0.97 to −0.79
6	−0.585	0.069	−8.52	<.005	−0.82 to −0.45
8	−0.109	0.042	−2.62	.01	−0.19 to −0.027
9	−0.115	0.119	−0.96	.34	−0.35 to 0.12
10	−0.104	0.047	−2.23	.03	−0.196 to −0.012
12	−0.31	0.07	−1.39	<.005	−0.45 to −0.17
13	0.28	0.047	5.81	<.005	0.18 to 0.37
16	−0.70	0.06	−11.7	<.005	−0.82 to −0.58
17	−0.05	0.04	−1.17	.24	−0.13 to 0.034
18	−0.36	0.04	−9.27	<.005	−0.44 to −0.29

### Insurance

A significant regression equation was found by fitting a univariate model to analyze the effect of insurance type on AOR. Table 4 presents the results of this model. It is important to note that this model uses Medicare A as the reference level, because it is the largest level within the data set, as seen in Multimedia Appendix 2. Thus, the results of all other insurance types are computed relative to Medicare A, which had an AOR-predicted probability of 94.9%.

By calculating the odds ratios in Table 4, we found that compared with Medicare A, the odds of a referral being accepted are as follows:

- Decreases by 21.6% (95% CI −0.287 to −0.20) when a patient holds managed care
- Decreases by 67.0% (95% CI −1.164 to −1.05) when a patient holds medicaid
- Decreases by 50.3% (95% CI −0.777 to −0.62) when a patient holds an insurance type of “other”
- Increases by 13.7% (95% CI −0.047 to 0.30) when a patient holds private insurance

The predicted probabilities of both referral-level characteristics can be seen in Multimedia Appendix 4.

**Table 4:** Logistic regression results for referral insurance type (reference level—Medicare A).

Insurance type	Coefficient	SE	*z* value	*P* value	95% CI
Intercept	2.93	0.016	188	<.005	2.90 to 2.96
Managed care	−0.244	0.022	−10.9	<.005	−0.287 to −0.20
Medicaid	−1.11	−0.028	−39.3	<.005	−1.164 to −1.05
Other	−0.70	0.04	−17.6	<.005	−0.777 to −0.62
Private	0.13	0.09	1.43	.15	−0.047 to 0.30

### Diagnosis and Insurance

To understand insurance type and diagnosis category when each is adjusted for the other, we created a multivariate logistic regression model using both variables. The detailed results are presented in Table 5. We found that while the other feature is held constant, the coefficient values within our model remained similar to and consistent with the results shown in Tables 3 and 4.

**Table 5:** Multiple logistic regression results for insurance and diagnosis types (reference levels—diseases of the circulatory system and Medicare A).

Feature level	Coefficient	SE	*z* value	*P* value	95% CI
Intercept	3.11	0.03	99.2	<.005	3.05 to 3.17
1	−0.19	0.05	−3.58	<.005	−0.285 to −0.09
2	0.078	0.081	0.96	.34	−0.082 to 0.24
3	−0.26	0.058	−4.52	<.005	−0.38 to −0.15
4	0.13	0.15	0.89	.38	−0.16 to 0.42
5	−0.67	0.05	−14.1	<.005	−0.77 to −0.58
6	−0.54	0.07	−7.78	<.005	−0.67 to −0.4
8	−0.11	0.04	−2.71	.007	−0.19 to −0.03
9	−0.07	0.12	−0.57	.57	−0.3 to 0.17
10	−0.13	0.05	−2.76	.006	−0.22 to −0.04
12	−0.32	0.07	−4.48	<.005	−0.46 to −0.18
13	0.9	0.054	16.6	<.005	0.8 to 1.008
16	−0.61	0.06	−10.2	<.005	−0.73 to −0.49
17	−0.06	0.043	−1.37	.17	−0.14 to 0.025
18	−0.37	0.039	−9.3	<.005	−0.44 to −0.29
Managed care	−0.24	0.024	−9.7	<.005	−0.29 to −0.19
Medicaid	−1.02	0.03	−32.4	<.005	−1.09 to −0.96
Other	−0.67	0.044	−15.3	<.005	−0.75 to −0.58
Private	0.31	0.099	3.08	<.005	0.111 to 0.5

### Regression Analyses—Facility Characteristics 5-Star Rating

By calculating the odds ratios of this model from Table 6, we found that, compared with 1-star facilities, the odds of a referral being accepted are as follows:

- Increases by 23.6% (95% CI 0.148-0.276) when a facility holds a 2-star rating
- Increases by 22.3% (95% CI 0.140-0.262]) when a facility holds a 3-star rating
- Increases by 8.6% (95% CI 0.022-0.144) when a facility holds a 4-star rating
- Increase by 38% (95% CI 0.253-0.391) when a facility holds a 5-star rating

**Table 6:** Logistic regression results for five-star rating (reference level—1-star facilities).

Feature level	Coefficient	SE	*z* value	*P* value	95% CI
Intercept	2.49	0.025	101.3	<.005	2.45-2.54
2.0	0.212	0.033	6.48	<.005	0.148-0.276
3.0	0.201	0.031	6.47	<.005	0.14-0.262
4.0	0.083	0.031	2.67	.008	0.022-0.144
5.0	0.322	0.035	9.17	<.005	0.253-0.391

### Geographic Area

Using rural facilities as our reference level, there was a 21.7% decrease in acceptance (95% CI −0.364 to −0.125) when a facility resides in an urban area versus a rural area (*P*<.005).

The predicted probabilities of both facility-level characteristics can be seen in Multimedia Appendix 5.

### Five-Star Rating and Geographic Area

By combining both 5-star rating and geographic area variables to fit a multivariate logistic regression model, we obtained similar results as for the individual univariate models of each predictor. Table 7 presents the results of this regression. When holding constant for overall 5-star rating, we found a 19.1% decrease in acceptance from facilities in urban settings rather than the 21.7% we were seeing previously. The coefficients correlated with overall 5-star ratings also remained fairly consistent with what we have seen in the univariate models, with only slight (<1.0%) variation in the log odds ratios.

**Table 7:** Multiple logistic regression results for overall 5-star rating and geographic area (reference levels—1-star facilities and rural areas).

Feature level	Coefficient	SE	*z* value	*P* value	95% CI
Intercept	2.7	0.065	41.4	<.005	2.58 to 2.83
2.0	0.21	0.033	6.6	<.005	0.15 to 0.28
3.0	0.197	0.031	6.33	<.005	0.136 to 0.26
4.0	0.081	0.031	2.61	.009	0.02 to 0.14
5.0	0.313	0.035	8.88	<.005	0.244 to 0.38
Urban	−0.21	0.06	−3.45	<.005	−0.332 to −0.092

## Discussion

### Overview

In this section, we have addressed the findings displayed in the analyses of daily operations, patient characteristics, and facility characteristics with regard to referral decisions. In addition, we have noted the limitations of this study, introduced plans for future work, and discussed our findings in relation to the role of leadership in the SNF setting.

### Daily Operations

Our univariate model analyzing occupancy looked at “low,” “medium,” and “high” occupancy tertiles. Point estimates for high occupancy had a slight decrease (6.9%) in the likelihood of an acceptance and low occupancy had a slight increase (6.7%) in the likelihood of an acceptance, relative to the “medium” category; however, we found no significant association between occupancy and AOR.

The results of our model analyzing the effect of nursing hours on AOR were positive point estimates, indicating increased AOR for a 1-hour per patient increase in CNA and LPN and a decreased AOR for a 1 hour per patient increase in RN. However, the CIs for these features were very close to 0, suggesting that nursing hours alone do not have a substantial impact on AOR.

### Patient Characteristics

In Table 3, we saw a number of primary diagnosis types that proved to be statistically significant with a threshold of *P*<.005. It is important to note that the primary diagnosis categories that were excluded were category 11 (complications of pregnancy; childbirth; and the puerperium), category 14 (Congenital Anomalies), and category 15 (Certain Conditions Originating in the Perinatal Period), as there were no data points in which the referral contained a diagnosis within those categories.

As we used category 7 (Diseases of the Circulatory System) as the reference category, all the coefficients displayed in Table 3 were compared with category 7. Thus, as most of the resulting coefficients were negative, they are evidently more likely to result in a denial compared with category 7. The diagnosis level most highly correlated with AORs was category 13 (Diseases of the Musculoskeletal System and Connective Tissue), with a log odds ratio of 31.7% (95% CI 0.183-0.368) compared with the reference category. This was followed closely by category 4 (Diseases of the Blood and Blood-Forming Organs) with a log odds ratio of 13.8% (95% CI −0.157 to 0.416) compared with the reference category. The remaining coefficients were all negative, demonstrating that, compared with our reference category, there is a negative mean change in AOR for every 1-unit increase in these diagnosis categories. The diagnoses with the largest negative mean change in AOR per 1-unit increase were category 5 (Mental Illness), category 16 (Injury and Poisoning), and category 6 (Diseases of the Nervous System and Sense Organs). These categories have a log odds ratio of 58.6% (95% CI −0.971 to −0.790), 50.24% (95% CI −0.815 to −0.581), and 44.3% (95% CI −0.719 to −0.450), respectively.

As categories 13, 7, and 4, the most commonly accepted diagnoses, contain a variety of diagnoses that typically result in a shorter stay, SNFs are often more inclined to accept these referrals. In addition, they contain a small percentage of diagnoses that require specialized equipment, meaning that most SNFs are already equipped to properly care for the patient. Conversely, categories 5, 16, and 6, the most commonly denied diagnoses, capture diagnoses that are notoriously challenging to manage. This means that the diagnosis may require specialized equipment, heightened attention and care, can be difficult to care for owing to behavioral issues, or may have an increased risk of injury owing to mobility issues. It is important to note that category 5 (Mental Illness) contains conditions such as dementia and similar illnesses correlated with brain function and brain disease.

We can see in Table 4 that the calculated coefficients are almost all negative compared with the reference category of Medicare A. The only insurance type with a positive difference is “private”; however, it is not statistically significant. In Table 5, we see that when holding the primary diagnosis constant, holding “private” increases the odds ratio of AOR by 37.9% (95% CI 1.13-1.67) and is statistically significant with *P*<.005.

Thus, we can reasonably conclude that although private insurance is rare (only 1.68% of all referrals, as shown in Multimedia Appendix 2), it is generally preferred. Private insurance is followed in desirability by medicare A, managed care, other, and medicaid. This means that it is significantly more likely for a referral from a patient holding medicaid to be denied versus a patient holding another type of insurance. One explanation for why we are seeing this ordering is that certain insurance types have a higher reimbursement rate and can thus be relied upon to cover most of the costs of a patient’s stay within the SNF. In addition, many facilities can only accept a subset of insurance types, resulting in immediate denials of referrals that do not hold a type in which the SNF can accept.

### Facility Characteristics

In our facility-level analysis, we fit 2 more univariate binary logistic regression models analyzing facility-level features of interest: overall 5-star rating and geographic area.

In the univariate model analyzing overall 5-star rating, we noted that the higher the facility rating (with the exception of 4-star facilities), the higher the likelihood of a referral being accepted. This indicates that higher-rated facilities are, on average, denying fewer of the referrals they receive. This may be because higher-rated facilities receive more appealing referrals from hospitals, have more diagnosis-specific equipment, and have better staffing.

Finally, in the multivariate model analyzing the effect of geographic area while holding overall 5-star rating constant, we noted that facilities located in urban areas are 19.1% more likely to deny a referral than those located in rural areas. A likely cause for this is the increased referral variation in urban areas. As facilities in urban areas are receiving referrals more regularly, the admission coordinators have more variety to choose from than those in rural areas who are often forced to accept patients that may be more “undesirable” to fill empty beds.

### Opportunities for SNFs

Recent policy changes regarding medicaid reimbursement for post–acute care services have been described as a “step in the right direction” to enable hospitals to more closely align post–acute care payment incentives with patient needs. Policy changes are expected to change where hospitals discharge medically complex patients as well as the services that patients may receive in post–acute care settings [4].

SNFs with excess capacity may have a financial incentive to increase occupancy [15], and a major motivation of this study is to create knowledge that SNFs can use to arrive at their appropriate occupancy by understanding and acting upon the patterns within their referral denials. We have described in detail the relationships of a multitude of referral- and facility-level features with AOR. In this section, we propose ways in which SNFs and their corresponding organizations could leverage this information to make informed and adaptive referral decisions.

When an SNF initially receives a referral from a hospital, there is a limited window of time in which to send a response if it wishes to maximize its chance of winning the referral. Thus, admissions coordinators within SNFs can only consider a subset of the personal health information within the referral data. Two primary pieces of personal health information that are considered by admissions coordinators are the primary diagnosis type and the insurance type of the patient being referred.

We first considered diagnosis type. Our analyses identified mental illness as the most commonly denied diagnosis category, followed by injury and poisoning and diseases of the nervous system and sensory organs. There may be opportunities for SNFs to accept these types of underserved patients. As these referrals are being denied more often by local competitors, there is a higher probability that if an SNF makes an offer on such referrals, they will win them and increase their occupancy, and if an SNF is regularly accepting and succeeding in caring for these types of referrals, they may be able to establish a reputation for providing quality care for such patients and thereby maintain or increase occupancy levels. If, because of a lack of physical equipment or resources, an SNF is unable to accept referrals that are common within less desirable categories (eg, dementia within mental illness), this may be something for the organization the SNF operates within to consider. For example, developing dedicated facilities that are designed to handle specific diagnoses (eg, memory care facilities for patients with dementia) may be worthwhile to consider if an organization is looking to expand and provide specialized care to an underserved population.

Next, we examine insurance types. Data show that medicaid enrollment increased amid the COVID-19 pandemic, growing by 17.7 million (24.9%) enrolled members from February 2020 to May 2022. During this time, all states in the United States experienced total medicaid enrollment growth ranging from 15.2% to 67.9% [16]. We concluded that medicaid was the least desirable insurance type compared with the others included in our analysis. With an increase in medicaid holders and a large number of SNFs preferring other types of insurance coverage, we suggest that, to leverage this information, SNFs reevaluate their payer mix and strive to accept an increased number of referrals holding medicaid. As they are being denied more often than any other type of insurance, it is statistically more likely for an SNF to win the lead. Furthermore, as stated earlier, the more a specific referral type an SNF accepts and succeeds in caring for (in this case, medicaid referrals), the more referrals of that type they are likely to receive from the hospital. It is important to note that along with medicaid, managed care is also increasingly being used throughout the health care system [17], and while it is accepted more often than medicaid, it is denied more than private insurance and Medicare A. Thus, facilities may want to aim to increase their percentage of managed care patients within their payer mix as well.

The effects of the abovementioned suggestions are amplified in urban areas, as there are significantly more referrals being sent from hospitals to SNFs in the immediate area. Therefore, each facility is likely to receive more referrals on average than they would in a rural area. While this also means that there are likely a greater number of SNFs as competition, if a substantial portion of this competition follows the denial patterns uncovered within this work, then it allows SNFs that are adjusting their referral decisions to accept “less desirable” referrals to increase occupancy.

It is of utmost importance for SNFs, if they are not already, to become purposeful with their own denials and be aware of the patterns of the surrounding SNFs. Failing which, the SNF may not behave as a team for which decisions are deliberately and rationally connected to the health needs of the specific populations and patients served. The adaptive challenges faced by SNFs require the mitigation of learning and organizational behavior changes.

### Future Work

This study acts as the initial assessment of a larger project aimed at using machine learning, specifically reinforcement learning, to assist facilities with multistakeholder sequential decision-making. This study delves into the patterns of referral decision outcomes to obtain an understanding of what factors facilities are most predominantly taking into account when they accept or deny a referral, giving decision makers insight into the common underlying attributes associated with their referral decisions. To create a machine learning model that can effectively aid health care professionals in decision-making processes, we must first understand why certain decisions are being made in the real world. This allows us to, when building decision support tools, ensure we meet the needs of decision makers. Using the data that we have been analyzing throughout this study, we plan to develop and implement an offline reinforcement learning methodology with the goal of developing a decision-making support tool that provides support for optimizing the decision patterns of different stakeholders within SNFs.

In addition, to enhance the analyses presented in this paper, we propose 2 prospective studies. First, a mixed methods study allowing researchers at multiple SNFs to collect data on losses and denials that may be recorded manually. Collecting otherwise uncaptured data and decision points surrounding these data could be fruitfully compared with the patterns discussed in this study’s findings. Second, a quantitative study that uses support vector machines, which use classification algorithms for 2-group classification problems, to determine which data points collected from SNFs not using an automated referral software can reasonably be included within our data set. This allows us to increase the number of total data points being used for our analyses (thus increasing the statistical soundness of results) while also ensuring that we are not introducing unnecessary bias to our data set.

Finally, we would like to explore more reliable methods of collecting daily operations data (in particular occupancy and nursing hours) to better understand the role of daily operations data in AOR decisions. This would involve putting into place a standardized practice of data recording (ie, logging the data at a particular time of day, each day) to ensure the consistency and quality of our data.

### Limitations

Our study is limited by the available data in 3 important ways. First, owing to data quality concerns, we filtered our data to include only the referrals sent to SNFs using an electronic referral software. The most major impact of this decision was a reduction in the proportion of rural facilities in the data, from approximately 1 rural facility referral for every 5 urban facility referrals to 1 rural facility referral for every 10 urban facility referrals, as rural facilities are less likely to implement electronic referral software. Future research focusing on rural facilities may require additional work to maintain data quality. It follows that there exists the possibility of selection bias, as SNFs using automated referral software may be larger in size, have more consistent staffing levels, and have more resources than those not using an automated referral software. Examining potential differences in referral processes, outcomes, and organizational characteristics would inform the extent to which our findings are subject to selection bias.

Second, some features were computed from values that were not automatically recorded (eg, calculating occupancy by dividing “current patients in facility” on a specified day by “total number of beds in facility” on that same day). This raised concerns about data quality; for example, if a number of patients were released from an SNF on a certain day, the “current patients in facility” count may not have been updated to reflect that decrease because the patients left after the count was documented, which in turn causes our “occupancy” value to be incorrect. Similarly, as RN, LPN, and CNA hours are summed over a 24-hour period and divided by the current patients within the facility at an unspecified time of day, we cannot be certain as to exactly how many nurses were active at the time of referral, causing the risk of our “nursing hours” value to be incorrect.

Third, because of data quality concerns, we were not able to include all relevant confounders in our model. Therefore, causal interpretations should be made with care. For future research initiatives, it will be important to consider how these important variables can be measured more reliably to better understand their impacts.

### Conclusions

Understanding referral patterns is an essential step in being more intentional in the process of accepting or denying these referrals. While a referral denial is not always a bad outcome from the point of view of the patient or SNF because the patient may not have been well served by that facility, we believe that many denials represent missed opportunities for SNFs to increase occupancy while maintaining a high standard of care.

Perhaps counterintuitively, facility occupancy and nursing hours at the time of referral plays a limited role in denial decisions based on our data. On the other hand, patient characteristics are highly informative: we can be confident that the referrals of patients insured with private insurance or medicare A are less often denied than the referrals of patients holding other insurance types. Further, the referrals of patients with diseases of the musculoskeletal system are less often denied than the referrals of patients within other diagnosis categories. Conversely, referrals of patients insured with medicaid are most often denied compared with all other insurance types, and referrals of patients with mental illness are most often denied compared with all other diagnosis categories.

We found a positive but nonmonotonic relationship between facility quality, as measured by a 5-star rating, and rates of accepting referrals, with the highest acceptance rates found among 5-star facilities. We hypothesize that these highest-rated facilities are receiving more appealing referrals from the hospital or are more likely to be a part of the hospital network, both of which may lead to more acceptances. We also found that facilities in urban areas had a 19.1% decrease in acceptance relative to their rural counterparts, which may stem from the wide variety of referrals that facilities in urban areas have to choose from.

We can conclude that some of the strongest drivers of denial are properties of the patient that they do not have control over. Understanding these patterns and considering ways to counteract them is a crucial component in developing strategies for SNFs to win more referrals and to maximize the opportunity for patients to receive the care they need.

## Appendix

Multimedia Appendix 1 Variation between referral decision outcomes for referrals received by skilled nursing facilities (SNFs) using automated software versus SNFs not using automated referral software.

Multimedia Appendix 2 Percent distributions of referral-level diagnosis and insurance types.

Multimedia Appendix 3 Percent distributions of facility-level overall 5-star ratings and urban versus rural status.

Multimedia Appendix 4 Predicted probabilities of referral-level diagnosis types and insurance types.

Multimedia Appendix 5 Predicted probabilities of facility-level overall 5-star ratings and urban versus rural status.

## Abbreviations

AOR: acceptance of referral
CNA: certified nursing assistant
ICD-10: International Classification of Diseases, 10th Revision
LPN: licensed practical nurse
RN: registered nurse
SNF: skilled nursing facility

## Data Availability

The data sets generated during and analyzed during this study are not publicly available because they are large, commercially available data sets. Despite this, a subset of the compiled data sets (daily operational data) is available from the corresponding author upon reasonable request.
