# Supplementary material for: Factors Influencing Admission Decisions in Skilled Nursing Facilities: Retrospective Quantitative Study
Source: J Med Internet Res. 2023 May 17;25:e43518. doi: 10.2196/43518 (PMC10233428; doi:10.2196/43518)
Supplement: Multimedia Appendix 1 [file jmir_v25i1e43518_app1.docx]

# Appendix A

**Table 9.** Variation between referral decision outcomes for referrals received by SNFs using automated software versus SNFs not using automated referral software.

SNFs Using Automated Referral Software

Percentage of Entire Data Set (%) 11.5 88.5

Percentage of Referrals Won (%) 76.7 82.7

Percentage of Referrals Lost (%) 16.8 12.0

Percentage of Referrals Denied (%) 6.5 5.2

SNFs Not Using Automated Referral Software
