# Supplementary material for: Factors Influencing Admission Decisions in Skilled Nursing Facilities: Retrospective Quantitative Study
Source: J Med Internet Res. 2023 May 17;25:e43518. doi: 10.2196/43518 (PMC10233428; doi:10.2196/43518)
Supplement: Multimedia Appendix 2 [file jmir_v25i1e43518_app2.docx]

# Appendix B

**Table 10.** Percent distribution of referral-level diagnosis types.

| Diagnosis | % of Total |
| --- | --- |
| 1 | 6.92 |
| 2 | 2.24 |
| 3 | 8.89 |
| 4 | 0.708 |
| 5 | 4.67 |
| 6 | 2.01 |
| 7 | 16.1 |
| 8 | 12.6 |
| 9 | 0.87 |
| 10 | 8.46 |
| 11 | - |
| 12 | 2.38 |
| 13 | 11.6 |
| 14 | - |
| 15 | - |
| 16 | 2.60 |
| 17 | 12.0 |
| 18 | 13.0 |

**Table 11.** Percent distribution of referral-level insurance types.

| Insurance Type | % of Total |
| --- | --- |
| Medicare A | 48.0 |
| Managed Care | 37.1 |
| Medicaid | 8.43 |
| Other | 4.76 |
| Private | 1.68 |
