# Supplementary material for: Factors Influencing Admission Decisions in Skilled Nursing Facilities: Retrospective Quantitative Study
Source: J Med Internet Res. 2023 May 17;25:e43518. doi: 10.2196/43518 (PMC10233428; doi:10.2196/43518)
Supplement: Multimedia Appendix 3 [file jmir_v25i1e43518_app3.docx]

# Appendix C

**Table 12.** Percent distribution of facility-level overall five-star ratings.

| Overall Five-Star Rating | % of Total |
| --- | --- |
| One-Star | 13.1 |
| Two-Star | 20.6 |
| Three-Star | 26.1 |
| Four-Star | 23.5 |
| Five-Star | 16.7 |

**Table 13.** Percent distribution of facility-level geographical areas.

Geographical Area % of Total

Urban 96.9

Rural 3.1
