# Supplementary material for: Factors Influencing Admission Decisions in Skilled Nursing Facilities: Retrospective Quantitative Study
Source: J Med Internet Res. 2023 May 17;25:e43518. doi: 10.2196/43518 (PMC10233428; doi:10.2196/43518)
Supplement: Multimedia Appendix 4 [file jmir_v25i1e43518_app4.docx]

# Appendix D

**Table 14.** Predicted probabilities of diagnosis types.

| Diagnosis | Predicted Probability (%) |
| --- | --- |
| 1 | 93.3 |
| 2 | 94.4 |
| 3 | 92.2 |
| 4 | 95.1 |
| 5 | 87.5 |
| 6 | 90.4 |
| 7 | 94.7 |
| 8 | 93.8 |
| 9 | 93.7 |
| 10 | 93.9 |
| 11 | - |
| 12 | 92.5 |
| 13 | 95.7 |
| 14 | - |
| 15 | - |
| 16 | 89.4 |
| 17 | 94.1 |
| 18 | 92.2 |

**Table 15.** Predicted probabilities of insurance types.

| Insurance Type | Predicted Probability (%) |
| --- | --- |
| Medicaid A | 94.9 |
| Managed Care | 93.6 |
| Medicaid | 86.1 |
| Other | 90.3 |
| Private | 95.5 |
