# Supplementary material for: Factors Influencing Admission Decisions in Skilled Nursing Facilities: Retrospective Quantitative Study
Source: J Med Internet Res. 2023 May 17;25:e43518. doi: 10.2196/43518 (PMC10233428; doi:10.2196/43518)
Supplement: Multimedia Appendix 5 [file jmir_v25i1e43518_app5.docx]

# Appendix E

**Table 16.** Predicted probabilities of facility-level overall five-star ratings.

Overall Five-Star Rating Predicted Probability (%)

1.0 94.1

2.0 94.9

3.0 94.8

4.0 94.2

5.0 95.3

**Table 17.** Predicted probabilities of geographical location.

Location Predicted Probability (%)

Rural 94.9

Urban 94.2
